# COVID-19 Vaccination in Patients with Inborn Errors of Immunity Reduces Hospitalization and Critical Care Needs Related to COVID-19: a USIDNET Report

**DOI:** 10.1007/s10875-023-01613-5

**Published:** 2024-04-05

**Authors:** John McDonnell, Kimberley Cousins, M. Elizabeth M. Younger, Adam Lane, Hassan Abolhassani, Roshini S. Abraham, Salem Al-Tamemi, Juan Carlos Aldave-Becerra, Eman Hesham Al-Faris, Alberto Alfaro-Murillo, Suzan A. AlKhater, Nouf Alsaati, Alexa Michelle Altman Doss, Melissa Anderson, Ernestina Angarola, Barbara Ariue, Danielle E. Arnold, Amal H. Assa’ad, Caner Aytekin, Meaghan Bank, Jenna R. E. Bergerson, Jack Bleesing, John Boesing, Carolina Bouso, Nicholas Brodszki, Diana Cabanillas, Carol Cady, Meghan A. Callahan, Roberta Caorsi, Javier Carbone, Maria Carrabba, Riccardo Castagnoli, Jason R. Catanzaro, Samantha Chan, Sharat Chandra, Hugo Chapdelaine, Zahra Chavoshzadeh, Hey Jin Chong, Lori Connors, Filippo Consonni, Oscar Correa-Jimenez, Charlotte Cunningham-Rundles, Katherine D’Astous-Gauthier, Ottavia Maria Delmonte, Yesim Yilmaz Demirdag, Deepti R. Deshpande, Natalie M. Diaz-Cabrera, Victoria R. Dimitriades, Rasha El-Owaidy, Gehad ElGhazali, Suleiman Al-Hammadi, Giovanna Fabio, Astrid Schellnast Faure, Jin Feng, James M. Fernandez, Lauren Fill, Guacira R. Franco, Robert W. Frenck, Ramsay L. Fuleihan, Giuliana Giardino, Jessica Galant-Swafford, Eleonora Gambineri, Elizabeth K. Garabedian, Ashley V. Geerlinks, Ekaterini Goudouris, Octavio Grecco, Qiang Pan-Hammarström, Hedieh Haji Khodaverdi Khani, Lennart Hammarström, Nicholas L. Hartog, Jennifer Heimall, Gabriela Hernandez-Molina, Caroline C. Horner, Robert W. Hostoffer, Nataliya Hristova, Kuang-Chih Hsiao, Gabriela Ivankovich-Escoto, Faris Jaber, Maaz Jalil, Mahnaz Jamee, Tiffany Jean, Stephanie Jeong, Devi Jhaveri, Michael B. Jordan, Avni Y. Joshi, Amanpreet Kalkat, Henry J. Kanarek, Erinn S. Kellner, Amer Khojah, Ruby Khoury, Cristina M. Kokron, Ashish Kumar, Kelsey Lecerf, Heather K. Lehman, Jennifer W. Leiding, Harry Lesmana, Xin Rong Lim, Joao Pedro Lopes, Ana Laura López, Lucia Tarquini, Ingrid S. Lundgren, Julieann Magnusson, Ana Karolina B. B. Marinho, Gian Luigi Marseglia, Giulia M. Martone, Annamaria G. Mechtler, Leonardo Mendonca, Joshua D. Milner, Peter J. Mustillo, Asal Gharib Naderi, Samuele Naviglio, Jeremy Nell, Hana B. Niebur, Luigi Notarangelo, Matias Oleastro, María Claudia Ortega-López, Neil R. Patel, Gordana Petrovic, Claudio Pignata, Oscar Porras, Benjamin T. Prince, Jennifer M. Puck, Nashmia Qamar, Marco Rabusin, Nikita Raje, Lorena Regairaz, Kimberly A. Risma, Elizabeth H. Ristagno, John Routes, Persio Roxo-Junior, Negin Salemi, Christopher Scalchunes, Susan J. Schuval, Suranjith L. Seneviratne, Ashwin Shankar, Roya Sherkat, Junghee Jenny Shin, Abeer Siddiqi, Sara Signa, Ali Sobh, Fabiana Mascarenhas Souza Lima, Kristen K. Stenehjem, Jonathan S. Tam, Monica Tang, Myrthes Toledo Barros, James Verbsky, Eleni Vergadi, Dayne H. Voelker, Stefano Volpi, Luke A. Wall, Christine Wang, Kelli W. Williams, Eveline Y. Wu, Shan Shan Wu, Jessie J. Zhou, Alexandria Cook, Kathleen E. Sullivan, Rebecca Marsh

**Affiliations:** 1 Pediatric Allergy and Immunology, Cleveland Clinic Children’s Hospital, 9500 Euclid Ave/R3, Cleveland, OH 44195 USA; 2 Clinical Immunology, Departments of Medicine and Pediatrics, Icahn School of Medicine at Mount Sinai, New York, NY USA; 3 Division of Bone Marrow Transplantation and Immune Deficiency, Cincinnati Children’s Hospital Medical Center, Cincinnati, OH USA; 4 Department of Pediatrics, University of Cincinnati College of Medicine, Cincinnati, OH USA; 5 Department of Biosciences and Nutrition, Karolinska Institute, Stockholm, Sweden; 6 Research Center for Immunodeficiencies, Pediatrics Center of Excellence, Children’s Medical Center, Tehran University of Medical Sciences, Tehran, Iran; 7 Department of Pathology and Laboratory Medicine, Nationwide Children’s Hospital, Columbus, USA; 8 Dept of Pathology, The Ohio State Univ Wexner College of Medicine, Columbus, USA; 9 Department of Child Health, Sultan Qaboos University Hospital, Muscat, Oman; 10 Allergy and Clinical Immunology, Hospital Nacional Edgardo Rebagliati Martins, Lima, Peru; 11 Department of Internal Medicine, King Fahad Specialist Hospital, Dammam, Saudi Arabia; 12 Department of Internal Medicine and Clinical Immunology, Hospital San Juan de Dios, San José, Costa Rica; 13 Department of Pediatrics, College of Medicine, Imam Abdulrahman Bin Faisal University, Dammam, Saudi Arabia; 14 King Fahd Hospital of University, Al-Khobar, Saudi Arabia; 15 Division of Allergy and Immunology, The Children’s Hospital of Philadelphia, Philadelphia, PA USA; 16 Division of Pediatric Allergy, Immunology, and Pulmonary Medicine, St. Louis Children’s Hospital, St. Louis, MO USA; 17 Division of Allergy Immunology Pulmonary and Sleep Medicine, Department of Pediatrics, Children’s Mercy Kansas City, University of Missouri-Kansas City, Kansas City, MO USA; 18 Immunology and Histocompatibility Unit, Hospital C. G. Durand, Buenos Aires, Argentina; 19 Department of Pediatrics, Division of Allergy and Immunology, Loma Linda Children’s Hospital, Loma Linda, CA USA; 20 Immune Deficiency-Cellular Therapy Program, Center for Cancer Research, National Cancer Institute, Bethesda, MD USA; 21 Division of Allergy and Immunology, Cincinnati Children’s Hospital Medical Center, Cincinnati, USA; 22 Department of Pediatric Immunology, Dr. Sami Ulus Maternity and Children’s Health and Diseases Training and Research Hospital, Ankara, Turkey; 23 Department of Internal Medicine, Louisiana State University Health Sciences Center, New Orleans, USA; 24 Laboratory of Clinical Immunology and Microbiology, NIAID, NIH, Rockville, MD USA; 25 Cincinnati Children’s Hospital Medical Center, Cincinnati, OH USA; 26 Immunology Department, Hospital Nacional de Pediatría Prof. Dr. Juan P. Garrahan, Buenos Aires, Argentina; 27 Department of Pediatric Immunology, Children’s Hospital, Lund University Hospital, Lund, Sweden; 28 Immunology Unit-Hospital Sor María Ludovica, La Plata, Argentina; 29 Community Medical Center, Missoula, MT USA; 30 Lake Erie College of Osteopathic Medicine, Elmira, USA; 31 Center for Autoinflammatory Diseases and Immunodeficiencies, IRCCS Istituto Giannina Gaslini, 16147 Genoa, Italy; 32 Immunology Department, Hospital General Universitario Gregorio Maranon, Madrid, Spain; 33 Department of Medicine, Fondazione IRCCS Ca’ Granda Ospedale Maggiore Policlinico, Milan, Italy; 34 Pediatric Unit, Department of Clinical, Surgical, Diagnostic and Pediatric Sciences, University of Pavia, Pavia, Italy; 35 Pediatric Clinic, Fondazione IRCCS Policlinico San Matteo, Pavia, Italy; 36 Section of Pulmonology, Allergy, Immunology and Sleep Medicine, Department of Pediatrics, Yale University School of Medicine, New Haven, CT USA; 37 Department of Clinical Immunology & Allergy, Royal Melbourne Hospital, Melbourne, VIC Australia; 38 Clinical Immunology, Montreal Clinical Research Institute, Université de Montréal, Montreal, Canada; 39 Immunology and Allergy Department, Mofid Children’s Hospital, Shahid Beheshti University of Medical Sciences, Tehran, Iran; 40 Division of Allergy and Immunology, UPMC Children’s Hospital of Pittsburgh, University of Pittsburgh School of Medicine, Pittsburgh, PA USA; 41 Department of Medicine, Dalhousie University, Halifax, NS Canada; 42 Centre of Excellence, Division of Pediatric Oncology and Hematology, Meyer Children’s Hospital IRCCS, Florence, Italy; 43 Pediatric Pulmonology and Immunology Research Group, Universidad Nacional de Colombia, Bogotá, Colombia; 44 Department of Pediatric Clinical Immunology and Allergy, University of Sherbrooke, Sherbrooke, QC Canada; 45 Division of Basic and Clinical Immunology, Department of Medicine, University of California, Irvine, CA USA; 46 Department of Pediatrics, Columbia University Medical Center, New York, NY USA; 47 Division of Allergy and Immunology, Department of Internal Medicine, University of South Florida Morsani College of Medicine, Tampa, FL USA; 48 Division of Allergy, Immunology and Rheumatology, Department of Pediatrics, University of California Davis Health, Sacramento, CA USA; 49 Pediatric Allergy, Immunology and Rheumatology Unit, Children’s Hospital, Ain Shams University, Cairo, Egypt; 50 Abu Dhabi and College of Medicine and Health Sciences, Sheikh Khalifa Medical City, Union71 - Purehealth, United Arab Emirates University, Al Ain, United Arab Emirates; 51 College of Medicine, Mohammed Bin Rashid University of Medicine and Health Sciences, Dubai, United Arab Emirates; 52 Hospital Sor María Ludovica, La Plata, Argentina; 53 Clinical Immunology, Department of Medicine at Icahn School of Medicine at Mount Sinai, New York, NY USA; 54 Department of Allergy & Clinical Immunology, Cleveland Clinic Foundation, Cleveland, OH USA; 55 University Hospitals, Cleveland Medical Centers, Cleveland, OH USA; 56 Division of Clinical Immunology and Allergy, Hospital das Clínicas, Faculdade de Medicina, Universidade de São Paulo, São Paulo, Brazil; 57 Division of Infectious Disease, Cincinnati Children’s Hospital Medical Center, Cincinnati, OH USA; 58 Division of Pediatric Allergy, Immunology and Rheumatology, Columbia University Medical Center, New York, NY USA; 59 Pediatric Section, Department of Translational Medical Science, Federico II University, Naples, Italy; 60 Division of Allergy & Clinical Immunology, National Jewish Health, Denver, CO USA; 61 Department of Neurosciences, Psychology, Drug Research and Child Health (NEUROFARBA), University of Florence, Florence, Italy; 62 National Human Genome Research Institute, National Institutes of Health, Bethesda, MD USA; 63 Pediatric Hematology and Oncology, Children’s Hospital, Western University, London, ON Canada; 64 Division of Allergy and Clinical Immunology – IPPMG, Universidade Federal Do Rio de Janeiro, Rio de Janeiro, Brazil; 65 Helen DeVos Children’s Hospital Division of Allergy and Immunology, Michigan State University College of Human Medicine, East Lansing, MI USA; 66 Division of Allergy and Immunology, Department of Pediatrics, Perelman School of Medicine at University of Pennsylvania, Children’s Hospital of Philadelphia, Philadelphia, PA 19104 USA; 67 Immunology and Rheumatology Department, Instituto Nacional de Ciencias Médicas y Nutrición Salvador Zubirán, Mexico City, Mexico; 68 Department of Pediatrics, Washington University School of Medicine, St. Louis, MO USA; 69 Department of Clinical Immunology and Stem Cell Bank, University Hospital Álexandrovska, Sofia, Bulgaria; 70 Starship Child Health, Auckland, New Zealand; 71 Department of Paediatrics: Child and Youth Health, Faculty of Medical and Health Sciences, University of Auckland, Auckland, New Zealand; 72 Clinical Immunogenomics Research Consortium Australasia, Sydney, Australia; 73 Department of Pediatrics, Caja Costarricense de Seguro Social, Hospital Nacional de Niños, San José, Costa Rica; 74 Advanced ENT & Allergy, Medford, NJ USA; 75 Pediatric Nephrology Research Center, Research Institute for Children’s Health, Shahid Beheshti University of Medical Sciences, Tehran, Iran; 76 Allergy Immunology Associates Inc., Allergy Immunology Fellowship Associate Program Director University Hospitals of Cleveland Medical Center, Cleveland, USA; 77 Division of Immunobiology, Cincinnati Children’s Hospital Medical Center, Cincinnati, OH USA; 78 Mayo Clinic Children’s Center, Pediatric and Adult Allergy and Immunology, Mayo Clinic, Rochester, MN USA; 79 Kanarek Allergy Asthma Immunology, Overland Park, KS USA; 80 Division of Allergy and Immunology, Cincinnati Children’s Hospital Medical Center and University of Cincinnati, Cincinnati, OH USA; 81 Department of Pediatrics, College of Medicine, Umm Al-Qura University, Makkah, Saudi Arabia; 82 Division of Allergy and Immunology, Department of Pediatrics, Nationwide Children’s Hospital and The Ohio State University College of Medicine, Columbus, OH USA; 83 Department of Pediatrics, University of Buffalo Jacobs School of Medicine and Biomedical Sciences, Buffalo, NY USA; 84 Division of Allergy and Immunology, Department of Pediatrics, Johns Hopkins University, Baltimore, MD USA; 85 Molecular Medicine, Cleveland Clinic Lerner College of Medicine, Cleveland, OH USA; 86 Department of Rheumatology, Allergy and Immunology, Tan Tock Seng Hospital, 11 Jalan Tan Tock Seng, Singapore, 308433 Singapore; 87 UH Rainbow Babies and Children’s Hospital, Case Western Reserve University, Cleveland, OH USA; 88 Unidad de Inmunología E Histocompatibilidad, Hospital Dr. Carlos G. Durand, Buenos Aires, Argentina; 89 Section of Pathological Anatomy and Histopathology, Polytechnic University of the Marche Region, 60020 Ancona, Italy; 90 Pediatric Infectious Diseases, St. Luke’s Children’s Hospital, Boise, ID USA; 91 USIDNET, Towson, MD USA; 92 University of Buffalo Jacobs School of Medicine and Biomedical Sciences, Buffalo, NY USA; 93 Center for Rare and Immunological Diseases, Hospital 9 de Julho - Rede DASA, São Paulo, Brazil; 94 Department of Pediatrics, Columbia University Irving Medical Center, New York, NY USA; 95 Division of Allergy and Immunology, Department of Pediatrics, Nationwide Children’s Hospital and The Ohio State University Wexner College of Medicine, Columbus, OH USA; 96 Allergy & Immunology, Keck School of Medicine of USC, Los Angeles, CA USA; 97 Pediatric Hematology-Oncology, Institute for Maternal and Child Health IRCCS “Burlo Garofolo,”, Trieste, Italy; 98 Department of Infection and Tropical Medicine, Newcastle Upon Tyne Hospitals National Health Service (NHS) Foundation Trust and Translational and Clinical Research Institute, Newcastle University, Newcastle Upon Tyne, UK; 99 Department of Pediatrics, University of Nebraska Medical Center, Children’s Hospital and Medical Center, Omaha, NE USA; 100 Division of Pediatrics, Allergy and Clinical Immunology, Hospital Infantil Universitario de San José, Bogotá, Colombia; 101 Department of Pediatrics, Children’s National Hospital, Washington, D.C. USA; 102 Department of Clinical Immunology and Allergology, Institute of Mother and Child Health, Belgrade, Serbia; 103 Pediatrics, Department of Translational Medical Sciences, Federico II University, Naples, Italy; 104 Pediatric Immunology and Rheumatology Department, Hospital Nacional de Niños “Dr. Carlos Sáenz Herrera,”, San José, Costa Rica; 105 Division of Allergy and Immunology and Blood and Marrow Transplantation, Department of Pediatrics, University of California San Francisco School of Medicine and UCSF Benioff Children’s Hospital, San Francisco, CA USA; 106 Division of Allergy and Immunology, Department of Pediatrics, Ann & Robert H. Lurie Children’s Hospital of Chicago, Northwestern University Feinberg School of Medicine, Chicago, IL USA; 107 Chief of Immunology Unit, Children’s Hospital “Sor María Ludovica, Buenos Aires, Argentina; 108 Division of Allergy Immunology, Department of Pediatrics, Cincinnati Children’s Hospital Medical Center, Cincinnati, OH USA; 109 Division of Pediatric Infectious Diseases, Mayo Clinic, Rochester, MN USA; 110 Department of Pediatrics, Medical College of Wisconsin, Milwaukee, WI USA; 111 Division of Immunology and Allergy, Department of Pediatrics, Ribeirão Preto Medical School, University of São Paulo, São Paulo, Brazil; 112 Immunodeficiency Research Center, Isfahan University of Medical Sciences, Isfahan, Iran; 113 Immune Deficiency Foundation, 7550 Teague Road, Hanover, MD 21076 USA; 114 Division of Allergy and Immunology, Stony Brook Children’s Hospital, Stony Brook, NY USA; 115 Department of Clinical Immunology, Nawaloka Hospitals, Colombo, Sri Lanka; 116 Section of Rheumatology, Allergy and Immunology, Department of Internal Medicine, Yale University School of Medicine, New Haven, CT USA; 117 Houston ENT and Allergy, Houston, TX USA; 118 Department of Pediatrics, Faculty of Medicine, Mansoura University Children’s Hospital, Mansoura University, Mansoura, Egypt; 119 Children’s Hospital Los Angeles, Los Angeles, CA USA; 120 Division of Pulmonary, Critical Care, Allergy, and Sleep Medicine, University of California San Francisco, San Francisco, USA; 121 Department of Paediatrics, Medical School, University of Crete, Rethymno, Greece; 122 Division of Allergy and Immunology, Department of Medicine, Washington University School of Medicine, St. Louis, MO USA; 123 Dipartimento Di NeuroscienzeRiabilitazioneOftalmologiaGenetica e Scienze Materno Infantili, University of Genoa, 16132 Genoa, Italy; 124 Section of Allergy Immunology, Department of Pediatrics, Louisiana State University Health and Children’s Hospital New Orleans, New Orleans, LA USA; 125 Section of Rheumatology, Department of Pediatrics, Children’s Hospital of Colorado, University of Colorado School of Medicine, Aurora, CO USA; 126 Division of Pediatric Pulmonology, Allergy and Immunology, Department of Pediatrics, Medical University of South Carolina, Charleston, SC USA; 127 Division of Pediatric Allergy and Immunology, Department of Pediatrics, The University of North Carolina at Chapel Hill, Chapel Hill, NC USA; 128 University Hospitals Cleveland Medical Center, Cleveland, OH USA; 129 Allergy and Immunology Associates Inc., Mayfield Heights, OH USA; 130 Johns Hopkins University School of Medicine, Baltimore, MD USA; 131 Department of Clinical Immunology & Allergy, The Royal Melbourne Hospital, Melbourne, Australia

**Keywords:** Immunodeficiency, Immunization, Viruses: respiratory diseases, Outcomes

## Abstract

**Background:**

The CDC and ACIP recommend COVID-19 vaccination for patients with inborn errors of immunity (IEI). Not much is known about vaccine safety in IEI, and whether vaccination attenuates infection severity in IEI.

**Objective:**

To estimate COVID-19 vaccination safety and examine effect on outcomes in patients with IEI.

**Methods:**

We built a secure registry database in conjunction with the US Immunodeficiency Network to examine vaccination frequency and indicators of safety and effectiveness in IEI patients. The registry opened on January 1, 2022, and closed on August 19, 2022.

**Results:**

Physicians entered data on 1245 patients from 24 countries. The most common diagnoses were antibody deficiencies (63.7%). At least one COVID-19 vaccine was administered to 806 patients (64.7%), and 216 patients received vaccination prior to the development of COVID-19. The most common vaccines administered were mRNA-based (84.0%). Seventeen patients were reported to seek outpatient clinic or emergency room care for a vaccine-related complication, and one patient was hospitalized for symptomatic anemia. Eight hundred twenty-three patients (66.1%) experienced COVID-19 infection. Of these, 156 patients required hospitalization (19.0%), 47 required ICU care (5.7%), and 28 died (3.4%). Rates of hospitalization (9.3% versus 24.4%, *p* < 0.001), ICU admission (2.8% versus 7.6%, *p* = 0.013), and death (2.3% versus 4.3%, *p* = 0.202) in patients who had COVID-19 were lower in patients who received vaccination prior to infection. In adjusted logistic regression analysis, not having at least one COVID-19 vaccine significantly increased the odds of hospitalization and ICU admission.

**Conclusion:**

Vaccination for COVID-19 in the IEI population appears safe and attenuates COVID-19 severity.

## Introduction

Coronaviruses are medium-sized, enveloped, positive-stranded RNA viruses named for their crown-like appearance under the electron microscope [1]. Viruses in this family played an important role in human health long before the COVID-19 pandemic, responsible for both nonspecific upper respiratory tract infections and specific viral syndromes like severe acute respiratory syndrome (SARS) and Middle East respiratory syndrome (MERS). In 2019, cases of coronavirus-induced pneumonia clustered in the Hubei Province of China. This started out as a local outbreak in Wuhan but soon spread internationally, developing into a global pandemic. The implicated virus was designated SARS-CoV-2, and the disease that virus caused was named COVID-19.

Clinically, COVID-19 infections can range from asymptomatic to life threatening. Several factors place infected patients at higher risk for poor outcomes, including increasing age, obesity, and chronic disease [2, 3]. Among the many chronic diseases that may lead to more severe infection are inborn errors of immunity (IEI), a collection of diseases which lead to immunodeficiency and immune dysregulation. The earliest survey of 94 patients with IEI observed 10% mortality and found that risk factors for severe disease in the general public also affected outcomes in patients with IEI [4]. A recently published review of the literature supports that mortality among most IEI diagnosis subgroups ranges from 4 to 16% [5]. One large collection reported that the case fatality rate could be as high as 100 times the general population among patients 0–19 years of age. Patients with disrupted type I interferon (IFN) immunity in particular have worse outcomes [6–9].

The first COVID-19 vaccines became available in 2020, and since their introduction, vaccines have proven to be critically important to decreasing both SARS-CoV-2 spread and COVID-19 disease severity. There have been obvious concerns regarding how well vaccines work in patients with IEI. Few studies have been performed, and the highly varied nature of the more than 450 IEI disorders make generalization across disorders impossible. IEI patients can have lower rates of seroconversion compared to healthy controls, though responses tend to improve with additional (third dose) vaccine administrations or when given in the setting of previous COVID-19 infection [5, 10–13]. In addition to antibody responses to COVID-19 vaccination, T-cell responses have proven to be important [14, 15]. Pham and colleagues found that most patients with IEI were able to mount at least a T-cell response to vaccination, even if their humoral immune response to vaccination was lackluster [16]. These findings suggested the importance of COVID-19 vaccination even in patients with severe humoral immune defects, as these patients may benefit from adaptive cellular immune responses even in the absence of a functioning humoral immune system.

Regardless of laboratory-observed vaccination responses, an important consideration for both IEI patients and practicing immunologists is the real-world effectiveness of vaccination against SARS-CoV-2. A previous large healthcare claims study of vaccinated patients found that most infections after vaccination resulting in hospitalization and/or death occurred in patients with primary and secondary immunodeficiencies, but no data were presented regarding just primary IEI patients [17]. A small single-center study of COVID-19 outcomes in 113 IEI patients who predominantly had CVID, hypogammaglobulinemia, and agammaglobulinemia observed that COVID-19-related hospitalization occurred in 40% of unvaccinated patients versus 4% in vaccinated patients, suggesting a dramatic protective effect of vaccination [18].

Overall, COVID-19 vaccines have proven safe in the general population, although mild local and systemic reactions are common such as pain, lymphadenopathy, headache, and fever. Although severe events are possible, including myocarditis, pericarditis, anaphylaxis, and thromboembolic events, these serious adverse events are rare [19, 20]. Little is known about the safety of COVID-19 vaccines in patients with IEI. For example, patients with autoinflammatory IEIs may fear that vaccination could precipitate a disease flare, requiring increased immunosuppression or hospitalization. Indeed, the rarity of many IEI conditions and the relative recency of COVID-19 disease has made it difficult for professional organizations, the normal adjudicators of such questions, to be able to determine if there are unique or increased risks for these patients. In fact, the 2021 consensus statement from the European Alliance of Associations for Rheumatology and the American College of Rheumatology on diagnosis and management of type 1 interferonopathies expresses agnosticism, stating “whether vaccines against COVID-19 have the potential to provoke a disease flare is unknown” and “there are currently no data to back specific recommendations” [21].

In this study, we investigated the real-world safety and effectiveness of vaccination in IEI patients. Our findings demonstrate that COVID-19 vaccination is safe and effective in a large, phenotypically diverse, and multinational IEI registry including more than 1000 patients.

## Materials and Methods

This study was performed as a collaboration between Cincinnati Children’s Hospital, the US Immunodeficiency Network (USIDNET), the Clinical Immunology Society, and additional physicians who contributed patient data. We created a COVID-19-specific registry database as part of the USIDNET for the collection of IEI patient data related to SARS-CoV-2 infection and/or SARS-CoV-2 vaccination. This REDCap database was used to house de-identified clinical patient data submitted by immunologists worldwide. The study was approved as exempt research by the Cincinnati Children’s Hospital institutional review board (IRB ID: 2021–0406). Members of the Clinical Immunology Society (CIS) were invited by email to contribute patient data via entry into the registry database. The database opened for entries on January 1, 2022, and closed on August 19, 2022.

Patients of any age with IEI and COVID-19/SARS-CoV-2 infection, COVID-19 vaccination, or both were eligible for inclusion. Diagnoses were linked to International Union of Immunological Societies (IUIS) subcategories by phenotypic or molecular defect as entered by the clinician [22]. All types of COVID-19/SARS-CoV-2 infections and complications were eligible for inclusion—asymptomatic, acute, long COVID, and multisystem inflammatory syndrome in children or adults (MIS-C/MIS-A). In addition to basic demographics and information on the nature of the subject’s IEI and relevant medical comorbidities, data were collected on hospitalization, requirement of ICU care, and patient survival. For patients who had COVID-19 vaccination, data were collected on vaccine side effects, need for escalation of IEI treatment in relation to vaccination, and healthcare utilization.

Primary outcomes of interest were (1) adverse vaccine effects and (2) real-world vaccine effectiveness in preventing hospitalization, ICU admission, and death. Adverse vaccine effects were determined by analyzing reported need for medical care in association with vaccination (emergency, outpatient, and inpatient environments), evaluating for significant changes to patients’ immunology medication regimen, examining for development of vaccine-induced myocarditis and anaphylaxis, and reviewing reported adverse effects beyond expected pain and fever for up to 3 days. Real-world vaccine effectiveness was assessed by comparison of reported hospital admission, ICU admission, and death in patients with at least one vaccine dose versus those without. Consideration of the timing of a COVID-19 infection in relation to vaccination was built into the analysis. For example, subjects who had a COVID-19 infection prior to vaccination were analyzed differently from subjects with first infection after vaccination.

We report descriptive statistics for the study population, including medians for continuous variables and counts and percentages for categorical variables. Categorical variables were compared using the Pearson chi-square test. Logistic regression analysis for outcomes of non-ICU hospitalization, ICU hospitalization, and death against vaccination status was performed both unadjusted and adjusted for potential confounding factors. Confounders considered in the adjusted models were age, obesity, kidney disease, lung disease, immunosuppressive medication use in the previous 3 months, neuromuscular disease, tracheostomy status, heart disease, sickle cell disease, and diabetes. Confounders that occurred infrequently in the data set (< 20 times each in the entire cohort)—namely, neuromuscular disease, tracheostomy, heart disease, diabetes, and sickle cell disease—were grouped together into a composite risk factor binary variable. For all these confounders, we assumed nonresponse on the survey instrument to be equivalent to a “no” answer, as not all fields were required for submission of a patient entry. All analysis was performed using Stata 17.0 (StataCorp. 2021. *Stata Statistical Software: Release 17*. College Station, TX: StataCorp LLC).

## Results

### Patients

A total of 1245 subjects were entered in the registry (Table 1). Of these, 806 (64.7%) had received at least one vaccine against COVID-19. The type of vaccine received was reported for 80.7% of vaccinated patients: the majority of patients received mRNA-based vaccines (84.0%) followed by viral vector (10.9%) and protein subunit vaccines (5.1%). Seven-hundred twenty-five received two or more vaccinations. Males were slightly predominant (53%), and patients were mostly Caucasian (70.1%) and from the USA (63.5%). Demographic characteristics were generally similar between the unvaccinated and vaccinated groups. Major exceptions were age (vaccinated patients tended to be older) and country of the treating medical center (higher percentage of vaccinated patients in US centers). The burden of comorbidities including lung disease, obesity, diabetes, and other conditions was similar between the groups, although the proportion of patients with history of bone marrow transplant (*n* = 96) was higher in the vaccinated group (8.9% compared to 5.5%).

**Table 1:** Demographic characteristics of subjects in the USIDNET registry

	Overall	≥ 1 vaccine	No vaccine
**Demographics**	1245	806 (64.7)	439 (35.3)
Sex			
Female	585 (47.0)	379 (47.0)	206 (46.9)
Male	660 (53.0)	427 (53.0)	233 (53.1)
Race/ethnicity*			
White	873 (70.1)	567 (70.3)	306 (69.7)
Black	57 (4.6)	34 (4.2)	23 (5.2)
Native American	3 (0.2)	3 (0.4)	0 (0.0)
Asian	31 (2.5)	19 (2.4)	12 (2.7)
Hawaiian	5 (0.4)	3 (0.4)	2 (0.5)
Other	91 (7.3)	52 (6.4)	39 (8.9)
Not Reported	185 (14.9)	128 (15.9)	57 (13.0)
Age at entry (years) median (p25–p75) (min–max)*	22 (14–43) (0–80)	28 (17–48) (4–80)	15 (8–26) (0–80)
Evaluating center			
US-based*	791 (63.5)	529 (65.6)	262 (59.7)
Comorbidities			
Lung disease^	440 (35.3)	290 (36.0)	150 (34.2)
Obesity^	154 (12.4)	107 (13.3)	47 (10.7)
Neuromuscular disease^	3 (0.4)	3 (0.4)	0 (0)
Tracheostomy^	6 (0.5)	3 (0.4)	3 (0.7)
Heart disease^	16 (1.3)	11 (1.4)	5 (1.1)
Sickle cell disease^	1 (0.1)	1 (0.1)	0 (0)
Diabetes^	10 (0.8)	7 (0.9)	3 (0.7)
Renal disease^	67 (5.4)	44 (5.5)	23 (5.2)
Immunosuppressive medications (past 3 months)^^	238 (19.1)	165 (20.5)	73 (16.6)
Bone marrow transplant history^	96 (7.7)	72 (8.9)	24 (5.5)

*Data are presented as no. (%) unless otherwise indicated*

^*^variable had missing data

^includes assumed variables—if entry was left blank assumed response was “no”

^^immunosuppressive medication as determined by physician entering data for patient

**Fig. 1:**
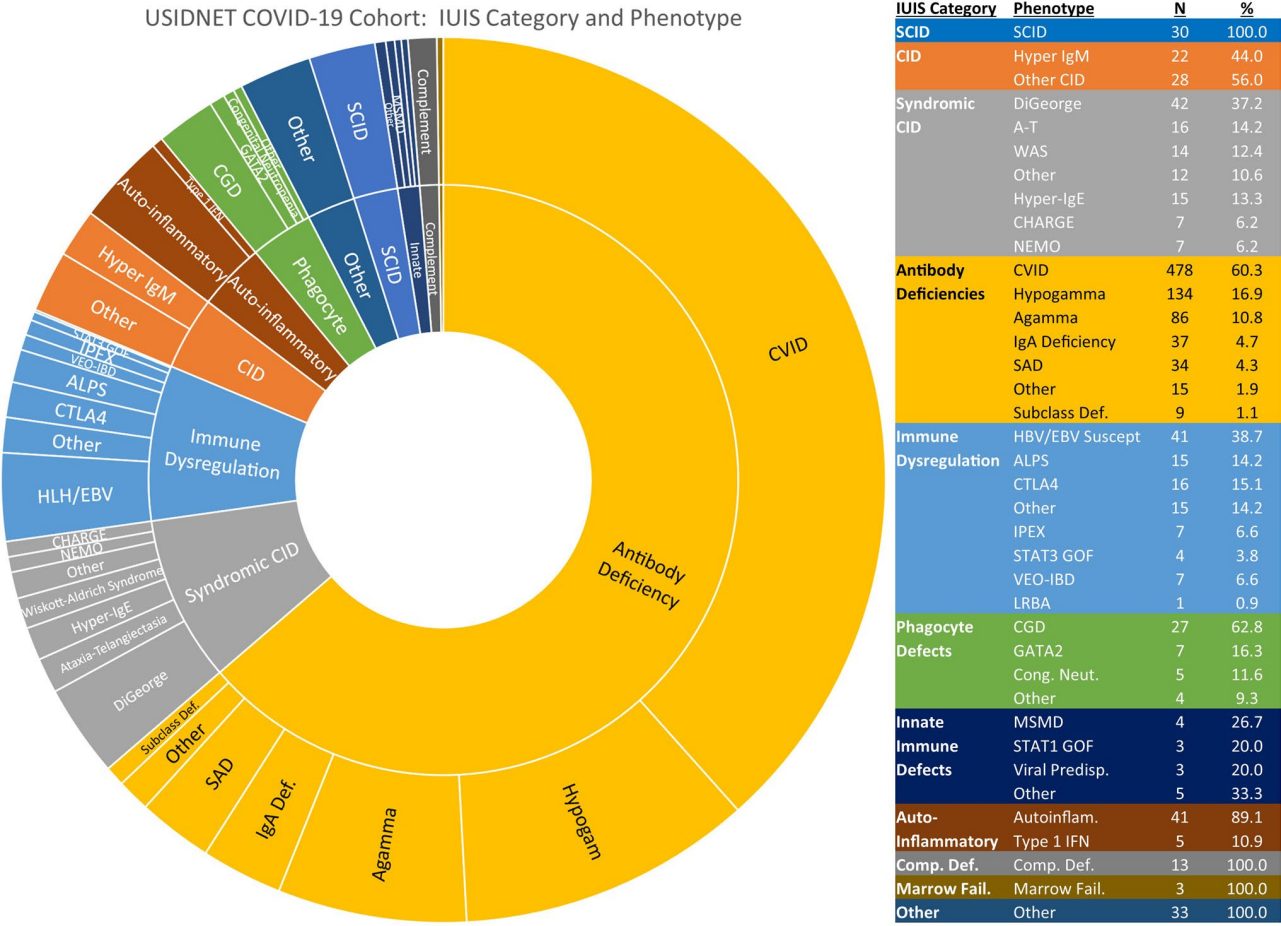
Patient diagnoses in USIDNET registry, categorized by International Union of Immunologic Societies (IUIS) schema. General IUIS categories further subclassified based on phenotype or genetic defect. Abbreviations: SCID, severe combined immune deficiency; CID, combined immune deficiency; A-T, ataxia-telangiectasia; WAS, Wiskott-Aldrich syndrome; CHARGE, coloboma/heart defects/atresia choanae/growth retardation/genital abnormalities/ear abnormalities; NEMO, nuclear factor-kappa B essential modulator deficiency; CVID, common variable immune deficiency; hypogamma, hypogammaglobulinemia; agamma, agammaglobulinemia; Comp. Def., complement deficiency; SAD, specific antibody deficiency; Subclass Def., IgG subclass deficiency; IgA Def., IgA deficiency; HLH/EBV Susc., hemophagocytic lymphohistiocytosis and EBV susceptibility; ALPS, autoimmune lymphoproliferative syndrome; IPEX, immune dysregulation/polyendocrinopathy/enteropathy/X-linked syndrome; VEO-IBD, very early onset inflammatory bowel disease; CGD, chronic granulomatous disease; MSMD, Mendelian susceptibility to mycobacterial disease; Cong. Neut., congenital neutropenia; Marrow Fail., bone marrow failure; Viral Predisp., predisposition to severe viral infection

A wide variety of IEI phenotypes and molecular diagnoses were represented in the cohort (Fig. 1). Most patients (*n* = 793, 63.7%) had antibody defects, predominantly CVID, hypogammaglobulinemia, and agammaglobulinemia. Combined immune deficiencies, syndrome-associated and otherwise, together made up the second largest category (*n* = 163, 13.1%). Disorders of immune dysregulation, including primary immune regulatory disorders and genetic disorders associated with hemophagocytic lymphohistiocytosis (HLH) or Epstein-Barr virus (EBV) susceptibility, were also well represented (*n* = 106, 8.5%). Forty-six patients had autoinflammatory disorders. There were 27 patients with chronic granulomatous disease (CGD) and 16 patients with other disorders of phagocyte function or number. Remaining categories are shown in Fig. 1. Note that 33 patients did not have enough information recorded to be categorized into a specific grouping. The breakdown of molecular diagnoses is given in Table 2 and included close to 150 different genetic diagnoses. The genetic disorders in the registry with 10 or more patients reported included pathogenic changes in *ADA*, *CD40L*, *ATM*, *WAS*, *BTK*, *TNFRSF13B*, *CTLA4*, *XIAP*, and 22q11 deletion.

**Table 2:** Molecular defects of subjects in the USIDNET COVID-19 registry as entered and categorized by registering clinicians

Category	Defect	*N* (%)	Category	Defect	*N* (%)	Category	Defect	*N* (%)	Category	Defect	*N* (%)
**SCID**	ADA	12 (40.0)	*(Antibody*	NFKB1	8 (1.0)	**Immune**	nr	38 (35.9)	**Autoinflammatory**	NLRP3	6 (13.0)
	nr	5 (16.7)	*Def. Cont)*	NFKB2	7 (0.9)	**Dysregulation**	CTLA4	16 (15.1)		ADA2	5 (10.9)
	RAG1	3 (10.0)		STAT3 GOF	4 (0.5)		XIAP	11 (10.4)		MEFV	4 (8.7)
	IL7R	2 (6.7)		47 + 21	3 (0.4)		FOXP3	5 (4.7)		MVK	4 (8.7)
	LIG4	2 (6.7)		CTLA4	3 (0.4)		AIRE—AR	4 (3.8)		IL1RN	3 (6.5)
	DCLRE1C	1 (3.3)		IGLL1	3 (0.4)		SH2D1A	4 (3.8)		PSTPIP1	3 (6.5)
	IL2RA	1 (3.3)		Kabuki	3 (0.4)		STAT3 GOF	4 (3.8)		nr	3 (6.5)
	IL2RG	1 (3.3)		NOD2	3 (0.4)		STXBP2	3 (2.8)		CARD14	2 (4.4)
	JAK3	1 (3.3)		16p11	2 (0.3)		TNFRSF6	3 (2.8)		CDC42	2 (4.4)
	NHEJ1	1 (3.3)		DUOX2	2 (0.3)		UNC13D	3 (2.8)		TMEM173	2 (4.4)
	RAG2	1 (3.3)		IKBKB	2 (0.3)		FAS	2 (1.9)		ATP6AP1	1 (2.2)
**CID**	nr	20 (40.0)		IKZF1	2 (0.3)		LRBA	2 (1.9)		COPA	1 (2.2)
	CD40L	12 (24.0)		IRF2BP2	2 (0.3)		AIRE—AD	1 (0.9)		NFKB1	1 (2.2)
	AICDA	6 (12.0)		LRBA	2 (0.3)		ELF4	1 (0.9)		IFIH1	1 (2.2)
	RAG1	2 (4.0)		PIK3R1—AD	2 (0.3)		IPEX	1 (0.9)		S124F	1 (2.2)
	CARD11	1 (2.0)		TCF3	2 (0.3)		LYST	1 (0.9)		NLRC4	1 (2.2)
	CARMIL2	1 (2.0)		47 XXY	1 (0.1)		MAGT1	1 (0.9)		PEPD	1 (2.2)
	CD70	1 (2.0)		ADA2	1 (0.1)		PRKCD	1 (0.9)		RELA	1 (2.2)
	IL2RA	1 (2.0)		ATM	1 (0.1)		RAB27A	1 (0.9)		TNFAIP3	1 (2.2)
	PGM3	1 (2.0)		BLNK	1 (0.1)		SLC7A7	1 (0.9)		TNFRSF1A	1 (2.2)
	PIK3R1	1 (2.0)		C1QA	1 (0.1)		STX11	1 (0.9)		TNFSF13	1 (2.2)
	RMRP	1 (2.0)		CARD11	1 (0.1)		TNFAIP3	1 (0.9)		TRNT1	1 (2.2)
	SASH3	1 (2.0)		CD21	1 (0.1)		TPP2	1 (0.9)	**Complement**	nr	8 (61.5)
	STK4	1 (2.0)		CD40	1 (0.1)	**Phagocyte**	nr	21 (48.8)	**Deficiency**	C1S	1 (7.7)
	TNFRSF13B	1 (2.0)		CXCR4	1 (0.1)	**Defects**	GATA2	7 (16.3)		C2	1 (7.7)
**Syndromic**	22q11	40 (35.4)		DNASE2	1 (0.1)		CYBB	6 (14.0)		C4A + C4B	1 (7.7)
**CID**	ATM	16 (14.2)		DNMT3B	1 (0.1)		NCF1	4 (9.3)		C5	1 (7.7)
	WAS	14 (12.4)		DOCK2	1 (0.1)		CYBA	1 (2.3)		CFI	1 (7.7)
	STAT3 LOF	13 (11.5)		ERCC2	1 (0.1)		FCGR3A	1 (2.3)	**Unknown** (*n* = 33)		
	CHD7	7 (6.2)		FANCA	1 (0.1)		HAX1	1 (2.3)			
	IKBKG (NEMO)	7 (6.2)		GATA2	1 (0.1)		JAGN1	1 (2.3)	*nr* not reported		
	nr	5 (4.4)		HYOU1	1 (0.1)		NCF4	1 (2.3)			
	KMT2D	4 (3.5)		IGHM	1 (0.1)	**Innate Immune**	IL17RA	3 (20.0)			
	TBX1	2 (1.8)		IRF7	1 (0.1)	**Defects**	STAT1 GOF	3 (20.0)			
	FOXI3	1 (0.9)		LIG4	1 (0.1)		IL12RB1	2 (13.3)			
	KMT2A	1 (0.9)		NCKAPIL	1 (0.1)		MYD88	2 (13.3)			
	NFKBIA	1 (0.9)		PIK3CG	1 (0.1)		IFNAR1	1 (6.7)			
	RMRP	1 (0.9)		PIK3R1—AR	1 (0.1)		IFNGR1—AD	1 (6.7)			
	SKIV2L	1 (0.9)		PLCG2	1 (0.1)		IFNGR1—AR	1 (6.7)			
**Antibody**	nr	633 (79.8)		POLG	1 (0.1)		TLR3	1 (6.7)			
**Deficiencies**	BTK	56 (7.1)		TCIRG1	1 (0.1)		nr	1 (6.7)			
	TNFRSF13B	17 (2.1)		TRNT1	1 (0.1)	**Bone Marrow**	DKC1	1 (33.3)			
	PIK3CD	9 (1.1)		TOP2B	1 (0.1)	**Failure**	SAMD9	1 (33.3)			
				WDR19	1 (0.1)		nr	1 (33.3)			

### Vaccine Complications

Adverse events reported after vaccination are listed in Table 3. Of the 806 patients who received at least one vaccine, only 17 were reported to seek medical care in the outpatient clinic (*n* = 9) or emergency room (*n* = 8) for a vaccine-related complication. One patient was hospitalized in association with the COVID-19 vaccine. This patient had a diagnosis of hyper-IgM syndrome and a history of recurrent cytopenias and developed an exacerbation of pre-existing autoimmune hemolytic anemia after his first COVID-19 mRNA vaccine.

**Table 3:** Vaccine safety in the IEI cohort (*n* = 806)

**Emergency room visit**			8 (1.0)	**Significant treatment changes**	10 (1.2)
**Outpatient clinic visit**			9 (1.1)	**Increased immunosuppression**	4 (0.5)
				IPEX patient with worsening eczema, required topical corticosteroids and dupilumab	
**Hospitalization**			1 (0.1)	STAT1 GOF patient developed severe aphthous ulcers, required baricitinib	
				Specific antibody deficiency patient with asthma exacerbation, required prednisone	
**Adverse effects/frequency** (some patients with > 1)			25 (3.1)	XIAP patient took previously prescribed oral steroids for possible disease flare	
	Fatigue	8		**Increased infections/requirement for antimicrobial treatment**	3 (0.4)
	Myalgia	5		CVID patient developed herpetic infections, required antiviral treatment	
	Arthralgia	3		MSMD patient developed *Salmonella* bacteremia and lymphadenitis	
	Headache	3		CVID patient started on antibiotic prophylaxis, unclear indication	
	Chest pain	2			
	Congestion	2		**Other**	3 (0.4)
	Diarrhea	2		Hereditary angioedema patient had increased disease flares requiring increased C1 INH	
	Palpitations	2		CVID patient developed cholelithiasis requiring surgery, 4 months after vaccine	
				Hyper IgM patient had exacerbation of pre-existing autoimmune hemolytic anemia	
	Abdominal pain	1			
	Confusion	1		**Anaphylaxis**	0 (0)
	Costochondritis	1			
	Cough	1		**Myocarditis**	0 (0)
	Diabetes	1			
	Fever (> 3 days)	1		**MIS-C/MIS-A**	1 (0.1)
	Flushing	1			
	Headache	1			
	Lethargy	1			
	Lymph node pain	1			
	Malaise	1			
	Nausea	1			
	Rash	1			
	Serum sickness	1			
	Sore throat	1			
	Unspecified	1			

Twenty-five patients were reported as having adverse effects secondary to the vaccines, with several having more than one listed complaint. Common issues included fatigue (8 patients), myalgia (5 patients), arthralgia (3 patients), and headache (3 patients). Most adverse effects were mild and self-limited, although one patient, a teenage girl with CVID, developed a serum sickness-like reaction after mRNA vaccination which required an outpatient physician visit and treatment with oral antihistamines. Seven patients required either increased immunosuppression or a change in antibiotic regimen after vaccination as detailed in Table 3. It is not clear what role vaccination may have played in many of these events, and some (e.g., onset of diabetes, gallstones) seem likely unrelated. There were no cases of anaphylaxis or vaccine-related myocarditis. MIS-C/MIS-A was reported to occur in one patient following vaccination. This patient had his second COVID vaccination, followed by a mild COVID-19 infection 6 days later. He then developed MIS-C approximately 6 weeks after the infection and required ICU care. His unusual presentation led to a genetic workup, and ultimately, this patient was diagnosed with familial HLH due to pathogenic variants in *STXBP2*.

### COVID-19/SARS-CoV-2 Infection

**Fig. 2:**
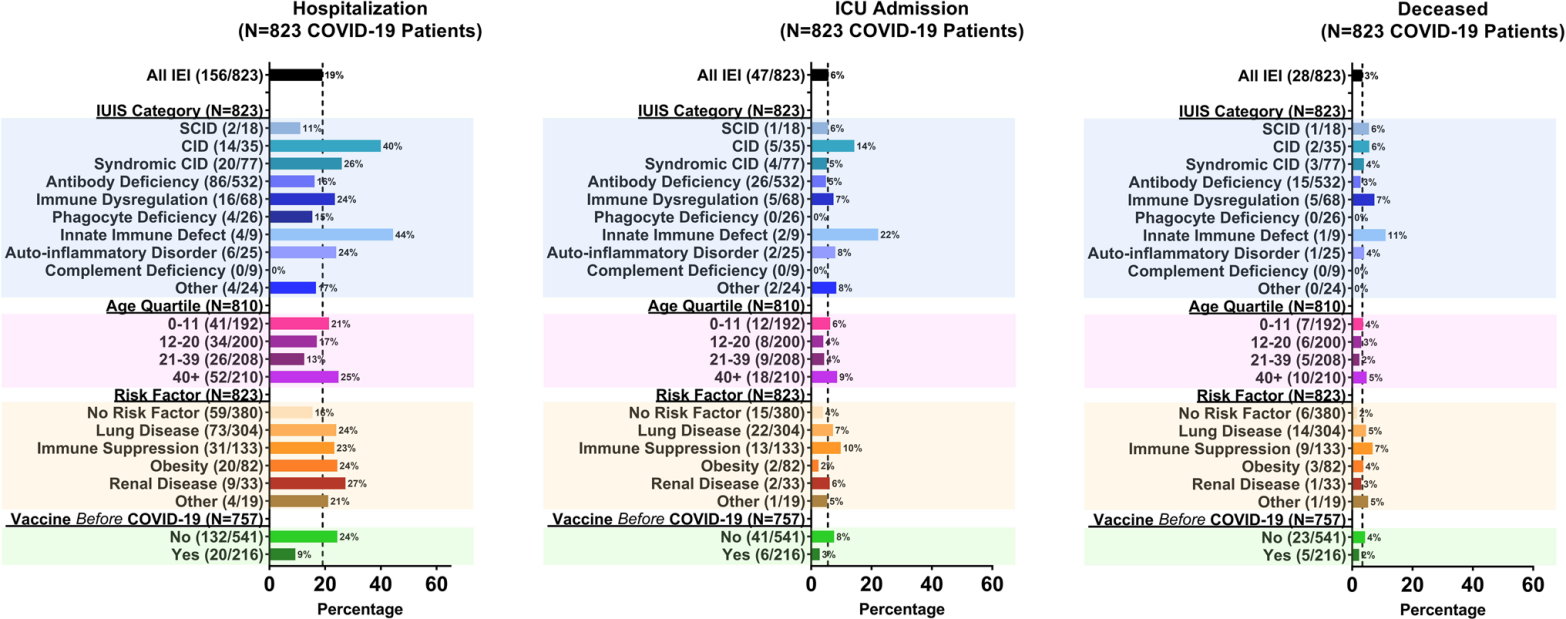
Hospitalization, ICU admission, and death among the USIDNET registry cohort. Categorization was adapted from International Union of Immunological Societies (IUIS) phenotypic classification. Age quartile (years) is based on patient age at time of COVID-19 infection. Three infected patients lacked data on age. COVID-19 risk factors included history of lung disease, immunosuppressive medication use in the 3 months preceding infection, obesity, and renal disease. Additionally, a measure of “other risk factors” was determined, representing a composite of uncommonly observed risk factors in the cohort—neuromuscular disease, tracheostomy, heart disease, sickle cell disease, and diabetes. Any patient with at least one of these uncommonly observed risk factors was counted for this measure. Vaccination was determined as receipt of at least one COVID-19 vaccine prior to SARS-CoV-2 infection. Sixty-six patients lacked adequate information on timing of vaccination relative to infection and were not included

Sixty-six percent (*n* = 823) of the patients in the USIDNET cohort experienced SARS-CoV-2 infections (Table 4). Of these, the majority (89.2%) were acute/symptomatic. MIS-C/MIS-A was reported in 7/823 patients (0.8%); these patients’ ages ranged from 2 to 24 years (median 11 years). The underlying IEI diagnoses in patients with MIS-C/MIS-A were X-linked agammaglobulinemia (*n* = 2), hyper-IgM syndrome (*n* = 2), interferonopathy (*n* = 1), inherited bone marrow failure (*n* = 1), and APECED (*n* = 1). Long COVID was reported in 13/823 patients (1.6%). One-hundred and fifty-one patients (18.4% of those infected) received monoclonal antibodies to prevent or treat COVID-19, and 41 (5.0% of those infected) received convalescent plasma. One-hundred and fifty-six IEI patients infected with SARS-CoV-2 required hospitalization (19.0% of those infected), 47 required ICU care (5.7%), and 28 died (3.4%) (Fig. 2). Characteristics of the 28 patients who died are given in Table 5. Most deceased patients had multiple comorbidities. The cause(s) of death for most adult patients included COVID-19, pneumonia, respiratory failure, acute respiratory distress syndrome (ARDS), or multiorgan failure. Sepsis was more commonly reported as an additional or only cause of death in pediatric patients, with *Escherichia coli* and *Stenotrophomonas maltophilia* identified in 2 patients.

**Table 4:** SARS-CoV-2 infection outcomes in the IEI cohort and effect of vaccination. Data are presented as column totals/percentages for outcomes of infection and hospitalization, ICU admission, and death among those infected

IEI patients with ≥ 1 SARS-CoV-2 infection		*N* = 823	*p*
Asymptomatic	64 (7.8%)		
Acute	734 (89.2%)		
MIS-C/MIS-A	7 (0.8%)		
Long	13 (1.6%)		
Other	5 (0.6%)		
**Required hospitalization for COVID-19/SARS-CoV-2 infection**		*N* = 156 (19.0%)	*p* < 0.001
History of ≥ 1 vaccine prior to hospitalization	20 (12.8%)		
No vaccination prior to hospitalization	132 (84.6%)		
Vaccinated but unknown timing relative to hospitalization	4 (2.6%)		
**Required ICU stay for COVID-19/SARS-CoV-2 infection**		*N* = 47 (5.7%)	*p* < 0.001
History of ≥ 1 vaccine prior to ICU	6 (12.8%)		
No vaccination prior to ICU	41 (87.2%)		
Vaccinated but unknown timing relative to ICU stay	0 (0%)		
**Died in association with COVID-19/SARS-CoV-2 infection**		*N* = 28 (3.4%)	*p* < 0.001
History of ≥ 1 vaccine prior to death	5 (17.9%)		
No vaccination prior to death	23 (82.1%)		
Vaccinated but unknown timing relative to death	0 (0%)

**Table 5:** Characteristics of deceased patients

Age	Diagnosis	Comorbidities and other conditions	Vaccination status	Reported c ause of death	Δ^
85	CVID	GLILD, CLL, ITP	Vaccinated	Acute hypoxic respiratory failure due to COVID pneumonia, septic shock	9
76	CVID	Obesity, chronic kidney disease, diabetes mellitus, coronary artery disease, GLILD	Unvaccinated	ARDS secondary to COVID-19	11
75	IgG subclass deficiency	Diabetes mellitus, AIHA	Unvaccinated	COVID pneumonia, ARDS, multiorgan failure	21
74	CVID	Asthma, obesity, atrophic gastritis, hypertension	Unvaccinated	COVID-19	6
74	CVID	Asthma, dysphagia, granulomatosis	Unvaccinated	Respiratory failure	10
74	CVID	Obesity, pulmonary granuloma	Vaccinated	COVID-19 pneumonia	14
54	CVID	ITP, bronchiectasis	Unvaccinated	Respiratory failure	21
50	CVID	Bronchiectasis, diarrhea, cirrhosis, portal hypertension	Vaccinated	Pneumonia, respiratory failure	9
49	CVID	Bronchiectasis	Vaccinated	Multiorgan failure	88
41	CVID	ITP, bronchiectasis, GLILD, lymphoproliferative disorder, portal HTN, lymphoma with secondary HLH	Unvaccinated	COVID-19 respiratory failure	12
39	Hypogammaglobulinemia	Kidney transplant, T-cell leukemia/lymphoma, Hodgkin lymphoma	Unvaccinated	Multiorgan failure, shock	35
28	Kabuki	Interstitial lung disease	Unvaccinated	Respiratory failure	26
28	Unspecified agammaglobulinemia	None	Vaccinated	Unknown	31
28	XLA	Bronchiectasis	Unvaccinated	ARDS due to COVID-19, bacterial pneumonia, sepsis	9
26	XLA	None	Unvaccinated	Respiratory failure	Unknown
18	KMT2A	Asthma, chronic respiratory failure on BiPAP, epilepsy, dysphagia	Unvaccinated	Respiratory failure	3
18	HLH (transplanted)	Encephalitis, hemolytic anemia, TMA	Unvaccinated	Pneumonia, COVID-19	50
15	APECED	Hypoparathyroidism, Addison disease, vitiligo, thyroiditis, asplenia, hypocalcemia	Unvaccinated	Pneumonia, pulmonary failure, nosocomial fungal sepsis	39
14	LRBA deficiency	Inflammatory bowel disease, AIHA, ITP, EBV, CMV colitis, arthritis, obliterative bronchiolitis, asthma	Unvaccinated	*E. coli* sepsis	Unknown
12	CVID	Malnutrition, bronchiectasis, TTP, diarrhea	Unvaccinated	Respiratory failure	36
12	STK4	Meningitis, cellulitis, ITP, AIHA, lymphadenopathy, seizures	Unvaccinated	Respiratory failure, cardiac failure	10
9	TPP2 deficiency	Ataxia, chronic idiopathic thrombocytopenic purpura	Unvaccinated	Respiratory failure, DIC	10
7	HLH (not transplanted)	None	Unvaccinated	HLH	87
3	Combined immune deficiency	Childhood bullous pemphigoid, candidiasis, failure to thrive	Unvaccinated	Sepsis	37
3	Unspecified autoinflammatory disorder	Psoriasis with arthropathy	Unvaccinated	Sepsis, ARDS	17
3*	IFNAR1 mutation	Chronic sinusitis, thrush, mucormycosis	Unvaccinated	Respiratory failure, left ventricular dysfunction/arrhythmias	51
1	NEMO (transplanted)	Norovirus enteritis, adenoviral gastroenteritis	Unvaccinated	*Stenotrophomonas sepsis*	76
< 1	SCID (not transplanted)	None	Unvaccinated	Sepsis, COVID-19 pneumonia	59

All vaccinated subjects had vaccine prior to acute infection; ages listed in years

^*^subject developed MIS-C in addition to acute COVID-19

^Δ = diagnosis date—death date, given in days

Hospitalization, ICU admission, and death rates varied by IUIS diagnosis group (Fig. 2). The highest rates were observed in patients with innate immune defects with hospitalization observed in 44%, ICU admission observed in 22%, and death observed in 11% of these patients. Patients with combined immune deficiencies, immune dysregulation, and autoinflammatory disorders also had higher rates of hospitalization, ICU admission, and death (Fig. 2). Lower rates were observed in patients with antibody deficiencies, and the lowest rates were observed in patients with phagocyte deficiencies and complement deficiencies, with no ICU admissions or deaths observed in those two patient groups. Rates of hospitalization, ICU admission, and death were higher in patients with comorbidities and in patients in the oldest age quartile (Fig. 2).

### Effect of Vaccination on COVID-19 Outcomes

Of 806 patients who received one or more vaccinations against SARS-CoV-2, 216 patients received vaccination *prior* to COVID-19/SARS-CoV-2 infection, 541 were vaccinated *after* infection, and timing of vaccination was not clear in the remaining patients. Patients who received vaccination after SARS-CoV-2 infection were counted in the unvaccinated group for analyses, and subjects with uncertain timing of vaccination relative to infection were excluded.

Rates of hospitalization, ICU admission, and death were all proportionately lower in the patients who received one or more vaccinations prior to COVID-19/SARS-CoV-2 infection (Fig. 2, Table 4). Twenty of 216 (9.3%) IEI patients who received one or more vaccinations prior to COVID-19 were hospitalized, compared with 132/541 (24.4%) who had not received at least one vaccine (*p* < 0.001). Six of 216 (2.8%) vaccinated patients were admitted to the ICU, compared with 41 of 541 (7.6%) unvaccinated patients (*p* = 0.013). Five of 216 (2.3%) vaccinated patients died, and 23 of 541 (4.3%) unvaccinated patients died (*p* = 0.202).

In unadjusted logistic regression analysis (Table 6), not having at least one COVID-19 vaccine prior to first COVID-19/SARS-CoV-2 infection significantly increased the odds of non-ICU admission by a factor of 3.16 (95% CI (1.92–5.22), *p* < 0.001) and the odds of ICU admission by a factor of 2.87 (95% CI (1.20–6.86), *p* = 0.018). Although the odds of death were also increased in the unvaccinated group, this difference was not statistically significant.

**Table 6:** Logistic regression analysis for COVID-19-related hospitalization, ICU admission, and death

	Unadjusted analysis		
	Odds ratio	95% CI	*p* value
**Outcome: hospitalization** (*n* = 757)*			
Variable			
No COVID-19 vaccine	3.16	1.92–5.22	< 0.001
**Outcome: ICU admission** (*n* = 757)*			
Variable			
No COVID-19 vaccine	2.87	1.20–6.86	0.018
**Outcome: death** (*n* = 757)*			
Variable			
No COVID-19 vaccine	1.87	0.70–4.99	0.209

	Adjusted analysis		
	Point estimate	95% CI	*p* value
**Outcome: hospitalization** (*n* = 748)^			
Variable			
No COVID-19 vaccine	3.84	2.28–6.49	< 0.001
Composite risk factors	1.08	0.34–3.46	0.901
Obesity	1.26	0.72–2.21	0.421
Renal	1.40	0.61–3.19	0.426
Immunosuppressive meds	1.37	0.85–2.20	0.196
Lung disease	1.55	1.06–2.25	0.023
Age	1.01	1.00–1.02	0.005
**Outcome: ICU admission** (*n* = 748)^			
Variable			
No COVID-19 vaccine	3.61	1.48–8.79	0.005
Composite risk factors	0.94	0.12–7.45	0.956
Obesity	0.30	0.07–1.28	0.103
Renal	0.76	0.17–3.41	0.719
Immunosuppressive meds	2.26	1.13–4.50	0.021
Lung disease	1.43	0.77–2.63	0.256
Age	1.02	1.00–1.03	0.011
**Outcome: death** (*n* = 748)^			
Variable			
No COVID-19 vaccine	2.30	0.85–6.28	0.103
Composite risk factors	1.63	0.20–13.09	0.645
Obesity	0.87	0.25–3.00	0.827
Renal	0.58	0.07–4.53	0.602
Immunosuppressive medications	2.69	1.17–6.19	0.020
Lung disease	1.59	0.73–3.45	0.239
Age	1.02	1.00–1.04	0.048

^*^Eight hundred twenty-three patients had a SARS-CoV-2 infection at least once. Of these, 66 patients were vaccinated but lacked information on timing of vaccine relative to COVID-19 illness and were thus excluded from unadjusted analysis

^Eight hundred twenty-three patients had a SARS-CoV-2 infection at least once. Of these, 66 patients were known to be vaccinated but lacked information on timing of vaccination relative to COVID-19 illness, and an additional 9 were missing data on age. These were excluded from adjusted analysis

Similarly, we performed regression analysis for the same outcomes, but adjusting for the potential confounders age, obesity, renal disease, immunosuppressive medication use, lung disease, and other composite risk factors (Table 6). Not having at least one COVID-19 vaccine prior to first COVID-19 infection significantly increased the odds of non-ICU admission (OR 3.84, 95% CI (2.28–6.49), *p* < 0.001) and ICU admission (OR 3.61, 95% CI (1.48–8.79), *p* = 0.005). While odds of death were increased in the nonvaccinated group (OR 2.30, 95% CI (0.85–6.28)), this difference was not statistically significant (*p* = 0.103. There was also a small but significant effect on the odds of hospitalization, ICU admission, and death for each increase in year of age (Table 6). Lung disease significantly impacted risk of hospitalization, and immunosuppressive medication use significantly impacted risk of ICU admission and death.

## Discussion

This is the largest registry report of COVID-19 vaccination and/or infection in IEI patients (*n* = 1245) to date. The disease burden in this multinational cohort of patients was diverse, with representation of even very rare diseases in each IUIS category.

Our study demonstrates that SARS-CoV-2 infections were most commonly mild in this phenotypically diverse patient population. Over 95% of patients can be expected to survive COVID-19. However, a significant proportion of infected IEI patients required hospitalization (19%) and ICU care (5.7%), and a minority did succumb (3.4%). The observed COVID-19 death rate in this large IEI registry cohort, which is largely US based, is higher than the US COVID-19 death rate in general (1.1%) and approaches that seen in medically underserved Ecuador (3.6%) [23]. Similar to previous studies, we observed that patients with innate immune defects, combined immunodeficiencies, disorders of immune dysregulation, and autoinflammatory disorders appear to have higher rates of severe complications of COVID-19 compared to patients with antibody deficiencies, phagocyte disorders, and complement deficiencies [4, 5, 24]. However, previous reports have included higher complication and death estimates in the IEI population than observed in our registry cohort. A recent systematic review on COVID-19 in patients with primary immunodeficiency found a case fatality rate of 9% and hospitalization rate of 49% [24]. These differences may be due to the different populations of the patients, with US patients representing a minority of patients in the review versus 63.5% of our USIDNET registry cohort. Abolhassani and colleagues performed a review of the COVID-19/IEI literature and found severe COVID-19 presentations in 21.5% of IEI patients and COVID-19-related mortality in 8.3% [7]. Of note, however, many of these cases of SARS-CoV-2 infection occurred prior to the widespread availability of vaccinations, and the Abolhassani cohort included more innate immune deficiencies which have been linked to more severe outcomes. A study based in the UK by Shields and colleagues found even higher rates of hospitalization (53.3%) and case fatality (39.2%), although this cohort included patients with secondary immune deficiencies in addition to IEI patients [25]. In contrast, an Italian IEI/COVID-19 study by Milito and colleagues reported a comparable infection mortality rate (3.8%) to that observed in our cohort [26] as did the Cousins study (3%) although the latter found a much larger difference in odds of hospitalization [18]. As before, the heterogeneity in these estimates is likely secondary to various confounding effects—temporal factors such as predominant SARS-CoV-2 variant at different times and increasing access to vaccination over time, cohort-level factors including diagnosis breakdown, and varying inclusion–exclusion criteria.

Vaccination in the IEI population was noted in our study to be quite effective in preventing COVID-19 general hospitalization and ICU admission, with > 3.5 times increased odds ratios for these outcomes in the unvaccinated group compared to those with at least one vaccine dose in adjusted regression analyses. Notably, the odds of death were not significantly decreased in the COVID-19 vaccinated group—a fact that is likely attributable to the generally low numbers of deaths in the cohort (28 patients), and even lower number of vaccinated deaths (5 patients), affecting statistical power.

Adverse effects of vaccination were generally mild and occurred in < 3.5% of vaccinated patients, further supporting the use of COVID-19 vaccination in patients with IEI. For the few vaccinated patients who required escalation of care after vaccination, it is not clear whether or how the vaccination event itself may have contributed to this. Importantly, there were no cases of anaphylaxis or vaccine-induced myocarditis in the cohort, though this does not rule out the occurrence. Myocarditis has been reported following a third mRNA vaccination in a 17-year-old male with CVID, for instance, and anaphylaxis would certainly be expected to occur in a minority of patients [27].

Limitations of the study include several inherent in registry-based observational research. These include recall bias on the part of clinicians filling out the survey, as well as ascertainment bias in terms of cases included. Additionally, respondents entered surveys at one point in time and were unable to update their entries later. Thus, information on repeat COVID-19 infections/outcomes and repeat COVID-19 vaccinations/outcomes was not captured. To facilitate analysis, subjects with more than one COVID-19 vaccine were pooled with those receiving only one, although it is reasonable to suppose that the degree of protection was different between these groups. Finally, the study design precluded evaluating for differences in rates of SARS-CoV-2 infection in vaccinated and unvaccinated patients. However, the apparent impact of vaccination on improving COVID-19 outcomes and the generally observed safety are important observations that may encourage hesitant IEI patients (up to 42%) to receive vaccination [28].

In summary, our study of a largely US-based registry cohort demonstrates that SARS-CoV-2 infections are mild in most patients with IEI but can be severe, and the percentages of serious COVID-19 outcomes (hospitalization, ICU care, or death) in this medically vulnerable group remain substantial. Vaccination appears safe and effective in decreasing serious outcomes among patients with diverse IEI.

## Data Availability

Researchers interested in access to the data may contact John McDonnell at mcdonnj@ccf.org.
